# Germination and Seedling Growth of Water Primroses: A Cross Experiment between Two Invaded Ranges with Contrasting Climates

**DOI:** 10.3389/fpls.2017.01677

**Published:** 2017-09-26

**Authors:** Morgane Gillard, Brenda J. Grewell, Caryn J. Futrell, Carole Deleu, Gabrielle Thiébaut

**Affiliations:** ^1^ECOBIO, UMR 6553 CNRS, Université de Rennes 1, Rennes, France; ^2^USDA-Agricultural Research Service Exotic and Invasive Weeds Research Unit, Department of Plant Sciences, University of California, Davis, Davis, CA, United States; ^3^IGEPP, UMR 1349 INRA, Université de Rennes 1, Le Rheu, France

**Keywords:** aquatic ecosystems, biodiversity, invasive plants, *Ludwigia* spp., climate warming, plant reproduction

## Abstract

Aquatic ecosystems are vulnerable to biological invasions, and will also be strongly impacted by climate change, including temperature increase. Understanding the colonization dynamics of aquatic invasive plant species is of high importance for preservation of native biodiversity. Many aquatic invasive plants rely on clonal reproduction to spread, but mixed reproductive modes are common. Under future climate changes, these species may favor a sexual reproductive mode. The aim of this study was to test the germination capacity and the seedling growth of two water primrose species, *Ludwigia hexapetala* and *Ludwigia peploides*, both invasive in Europe and in the United States. We performed a reciprocal transplant of seeds of *L. hexapetala* and *L. peploides* from two invasive ranges into experimental gardens characterized by Oceanic and Mediterranean-type climates. Our results showed that higher temperatures increased or maintained germination percentages and velocity, decreased survivorship of germinants, but increased their production of biomass. The origin of the seeds had low impact on *L. hexapetala* responses to temperature, but greatly influenced those of *L. peploides*. The invasiveness of water primroses in ranges with Oceanic climates might increase with temperature. The recruitment from seed banks by these species should be considered by managers to improve the conservation of native aquatic and wetland plant species.

## Introduction

Invasive plants represent a serious threat to the native biodiversity of inland aquatic ecosystems throughout the world. The damages caused by these non-native species are mostly linked to their high biomass, and their presence modifies water quality, hydrology (Strayer, 2010), or the composition and structure of native communities (Michelan et al., 2010; Stiers et al., 2011). Indeed, the presence of some invasive macrophyte species has negative effects on native macrophyte richness and functional diversity (Michelan et al., 2010). Invasive macrophytes can also profoundly modify food web structure through reduction and modification of the abundance and taxonomic composition of species in other trophic levels such as aquatic invertebrates (Stiers et al., 2011) and fishes (Gallardo et al., 2016). Exotic aquatic species have been shown to have proportionally larger ecological and economic impacts than exotic species in terrestrial ecosystems (Vilà et al., 2010). This suggests that there are fundamental differences in the colonization dynamics of aquatic ecosystems, and further demonstrates the vulnerability of these systems to invasion. The ability of exotic macrophytes to transform ecosystems (Yarrow et al., 2009; Evangelista et al., 2014) raises concerns about the future of native species, as some regions support many rare or threatened macrophyte species (Nicolet et al., 2004). Moreover, projected global climate changes are expected to magnify the negative impacts of invasive species with severe consequences for native species and ecosystems (Mainka and Howard, 2010). For example, recent studies suggest that warmer thermal conditions will result in a shift from native to exotic aquatic plant dominance (Hussner, 2014), and dominance of exotic macrophytes can modify the species composition of the standing vegetation and the persistent soil-stored seed bank by depleting the seed bank of native species (Vojtkó et al., 2017).

The colonization dynamics of aquatic plant species are poorly understood in the context of global climate change. Rises in temperature, CO_2_ concentrations, and precipitation changes directly and indirectly affect the phenology, productivity, and distribution of aquatic vegetation (Heino et al., 2009; Peeters et al., 2013). Furthermore, temperature can also influence seed character, germination processes, and germination rates (Leck and Brock, 2000). There is a need to understand key biological processes, including critical stages in the life cycle of invasive plant species, to predict responses of invasive species to climate warming. Under current environmental conditions, the primary mode of reproduction of invasive macrophytes is dispersal of vegetative fragments, even though sexual reproduction is possible. Some species such as the water primroses *Ludwigia hexapetala* (syn. *L. grandiflora* subsp. *hexapetala*) and *Ludwigia peploides* subsp. *montevidensis* (hereafter *L. peploides*) invest significant resources to rely on sexual reproduction, and this mode of reproduction is occasionally detected in *L. hexapetala* (Okada et al., 2009). These two *Ludwigia* species, native to South America, have widely colonized aquatic ecosystems in western Europe and in the United States (Wagner et al., 2007; Hussner, 2012). Their high biomass production generates high environmental impacts and economical costs, and their monospecific stands outcompete indigenous macrophytes (Dandelot et al., 2005; Thouvenot et al., 2013). For instance, the presence of *L. hexapetala* in Belgium affects the abundance of *Alisma plantago-aquatica, Ceratophyllum demersum*, and *Lycopus europaeus*, native to Europe (Stiers et al., 2011). In France *L. peploides* is self-compatible and usually fructiferous, whereas *L. hexapetala* has variable capsule production, and some populations are sterile (Dandelot et al., 2005). Sterile populations have not been observed in California. The two water primroses differ in ploidy levels: *L. hexapetala* is decaploid (2n = 80) and *L. peploides* is diploid (2n = 16). Seeds of these two closely related invasive *Ludwigia* species of varying ploidy levels may respond quite differently to environmental conditions. Polyploid populations of the wetland species *Mimulus guttatus* germinate over a larger range of temperatures than those from diploid populations (Vickery, 1967), and polyploid seeds of *Taraxacum* lineages from Japan germinated at higher percentages than diploids at optimal temperatures (Hoya et al., 2007). Seed germination is a crucial step that conditions the production of sexual propagules and the process widely depends on environmental conditions, including moisture, light, and temperature (Walck et al., 2011). Gioria and Pyšek (2017) recently highlighted the importance of studying germination responses of invasive species to climate changes, with the aim to develop sustainable control strategies of invasive species and restoration measures which favor germination of seed of desirable native species.

Plant populations exposed to changing environmental conditions and extreme events increase their chance of regeneration when they combine both asexual and sexual reproduction (Silvertown, 2008; Li, 2014). Thus, future climate change may alter the reproductive strategies of water primroses, promoting greater sexual reproduction, and survival of the species as an adaptation to climate conditions (Li, 2014). Furthermore, field observations in France showed that over the past decade the two species have produced more capsules than previously observed (Ruaux et al., 2009; Haury et al., 2014). The effectiveness of the sexual reproduction of water primroses is relatively little known, but may represent a threat in their invasive ranges due to rising temperatures. In addition, in their invasive ranges *Ludwigia* spp. are predicted to increase their potential distribution under future climate scenarios (Gillard et al., 2017a). Higher germination percentages and seedling survival under higher temperatures could allow *L. hexapetala* and *L. peploides* to reach uncolonized favorable environments and thereby to increase their future potential distribution. Therefore, understanding the germination dynamics of invasive *Ludwigia* species with rising temperatures is important for conservation management of the invaded ecosystems. In a previous study, we first explored germination responses of these species to a range of controlled temperatures in growth chambers (Gillard et al., 2017b). We showed that some populations had high germination percentages, and that germination capacity will be maintained with 3°C temperature warming. To further explore this process, in the subsequent study reported here, we conducted outdoor mesocosm experiments at two locations with contrasting temperature and other ambient climate conditions in the invaded ranges of France and California, with seed collected from eight naturalized populations of two *Ludwigia* within the two ranges. The objective of this current work was to compare the differences in germination dynamics and in survivorship of germinants when exposed to contrasting temperatures, as well as the impact of seed origin on performances. We hypothesized that (1) seed germination and seedling performances will be favored under hotter climatic conditions, (2) seed germination and performance of seedlings will be different depending on their provenance, and (3) due to its polyploid trait, *L. hexapetala* will outperform *L. peploides* for the tested variables.

## Materials and Methods

In this experiment, we aimed to test the effect of warmer climate on the germination capacities and seedling growth of two invasive water primroses. To do so, we examined a total of eight populations (four for each species) with seeds from two invaded ranges with contrasting climates.

### Study Sites: Two Invaded Ranges

The climate between the two ranges tested in this experiment is quite different at invasive *Ludwigia* population sites. The Russian River Watershed, in coastal northern California, is dominated by a temperate Mediterranean-type climate, with dry and hot summers (see Supplementary Figure S1). Impacted watersheds in northwestern France experience a temperate Oceanic climate, with warm summers but without a dry season (Supplementary Figure S1). According to Köppen climate classification, the two climate types are categorized as Csa and Cfb, respectively. The two regions are also characterized by their difference in latitude, which generates a slight difference of day length from 0.5 to 1 h between the experimental sites from May to July.

### Capsule Collection

Capsules were collected between August and October 2015 following maturation of seed, but before capsule dehiscence. Two naturalized populations of each of the two *Ludwigia* taxa were sampled from the Loire Valley of northwestern France (Loire River Watershed; **Table 1**), and an additional two naturalized populations of each taxa were sampled in Russian River and Napa River Watersheds in northern California (**Table 1**). In France, the four sites where capsules were collected are oxbows located on the Loire River, separated by 3–10 km. In California, the two collections sites of *L. hexapetala* in the Russian River were separated by 70 km; and seeds of *L. peploides* were collected in an impounded tributary of the Russian River and adjacent Napa River Watershed. In both invaded ranges, collection sites were characterized by dense vegetation beds of water primroses with no co-occurrence of the two species. We considered each sampled site as a population in this study, as the sites were discrete, widely separated, and locally the species experienced different conditions among sites. Seed capsules were collected from individuals separated by at least 10 m in population patches where possible, to ensure collection from distinct individuals. A maximum of three fruits were collected per primary erect stem of each sample plant, and were pooled with other capsules from the same population. Capsules were dried at ambient temperature, and then stored at 4°C in the dark. On average, they were 42 ± 17 seeds per capsule.

**Table 1:** Location of the sites where capsules were collected.

Species	Site code	Name of waterbody—site name	GPS coordinates	Climate
*L. hexapetala*	CALH1	Laguna de Santa Rosa tributary to Russian River—Laguna Ranch	38.446701, -122.836557	Mediterranean-type
	CALH2	Russian River—Asti	38.763413, -122.966920	
	FRLH1	Loire River—Ile du château	47.316483, 0.413346	Oceanic
	FRLH2	Loire River—Les Raguins	47.329167, 0.462044	
*L. peploides* subsp. *montevidensis*	CALP1	Sage Creek tributary to Napa River	38.490237, -122.347488	Mediterranean-type
	CALP2	Santa Rosa Creek tributary to Russian River	38.459315, -122.654070	
	FRLP1	Loire River—Ile Joli Coeur	47.325036, 0.437465	Oceanic
	FRLP2	Loire River—Port de Vallières	47.386191, 0.605725	

*The first two letters of the site code correspond to the area of origin (CA, California; FR, France) and the two last letters indicate the species (LH, *Ludwigia hexapetala*; LP, *Ludwigia peploides* subsp. *montevidensis*).*

### Experimental Design

An outdoor mesocosm experiment to evaluate germination responses of eight invasive *Ludwigia* populations was implemented simultaneously in experimental gardens at the USDA-ARS Aquatic Weed Research Facility at the University of California, Davis and at the ECOBIO Research Facility at the University of Rennes 1 from May through June 2016, concurrent with the primary timing of germination within natural populations at field sites. The capsules used were those collected the previous autumn. Before the beginning of the experiment, capsules were stored at 4°C in water for 10 days to mimic natural vernalization in humid conditions. In each experimental garden location, 12 capsules from each population were dissected, and a maximum of 24 seeds per capsule were kept for the experiment. We only used seeds that appeared to have fully developed, healthy-looking embryos. A total of 2304 seeds were buried individually, at 1 cm depth in Leach “Cone-tainers”^TM^ (Stuewe and Sons, Tangent, OR, United States) filled with a 9:1 (v/v) mixture of sand and potting soil (NPK 9–5–7). The cone-tainers pots were 14 cm high, with a diameter of 4 cm at the top, and holes at the bottom. Mesh screens were placed at the bottom of the pots to prevent soil mixture escape but also to allow its contact with water. Pots were arranged in trays of 98 with each tray containing the seeds from four capsules, and half-randomly placed in three to four tanks (dimensions: 2 m × 1.5 m or 1.95 m × 1.21 m) filled with 12.5 cm of tap water. Pots were maintained vertically with the bottom 6.5 cm centimeters of the pots immersed in water. The temperatures of water, air, and soil mix were measured hourly for the duration of the experiment (6 weeks). Air temperature and water temperature were recorded respectively with HOBO^®^ Pro v2 logger and HOBO^®^ U22 Water Temp Pro v2 in California, and with a station Davis Instrument Vantage Pro2 and SWS Mini-Diver in France. Soil mix temperatures were recorded with iButtons^®^ (type DS1922L and DS1921G, Maxim Integrated Products, Inc) buried in cone-tainers at the same depth as the seeds.

Germination of seeds and seedling survival were monitored three times a week for 6 weeks. Seedlings were considered emerged when a cotyledon was visible at the soil surface. The mean time to germination (MTG) was calculated to describe germination pattern, using the following equation (Luque et al., 2013):

$$\text{MTG}=\frac{\sum nD}{N}$$

where *n* is the number of seeds which germinated on day *D, D* is the incubation period until counting, in days, and *N* represents the total number of seeds that germinated during the experiment. After 46 days, the length of shoots and roots and the number of branches produced were measured. Shoots and roots were dried separately at 70°C for 72 h, and then weighed. Un-germinated seeds were extracted from the soil mix, and their viability was tested with a tetrazolium (2,3,5-triphenyl-2H-tetrazolium chloride) solution at 0.1% (Porter et al., 1947). Seeds were cut in half to bisect the embryo, and submerged in the tetrazolium solution for 48 h at 4°C. Viable embryos presented a pink or red color.

### Statistical Analyses

Statistical analyses were performing using statistical R^TM^ 3.2.3 software (R Development Core Team, 2015) with packages car (Fox and Weisberg, 2011), MuMIn (Barton, 2016), lme4 (Bates et al., 2015), AICcmodavg (Mazerolle, 2016), and mixtools (Benaglia et al., 2009). We applied linear models with mixed effects on the MTG, with species, climate, and seed provenance (i.e., invasive range) as fixed effects, and population as random effect. Binomial data such as seed germination, embryo viability, and seedlings survivorship were tested with generalized linear model with mixed effects, species, climate, and seed provenance as fixed effects, and population as random effect. Linear models with mixed effects were used to test the effect of seedling age, species, climate, seed provenance, and the interactions of seedling age, species, and seed provenance with climate (fixed effects), on seedling characteristics. The effects of capsules nested in tank and of populations were included in the model as random effects. Plots were generated from prediction of the linear models for seedling age by species and by range. Confidence intervals around the average predicted per seedling age were calculated as following: +1.96^∗^ standard deviation and -1.96^∗^ standard deviation. Variables were considered significantly different when the confidence intervals of the values predicted for the two climates were not overlapping. For both generalized linear models and linear models, the impact of fixed effects was tested with an analysis of deviance performed on the model. Moreover, the variability explained by the model was calculated for fixed and random effects according to the method developed by Nakagawa and Schielzeth (2013).

## Results

### Temperature Differences between Experimental Sites

During the experiment, the average soil temperature in the cone-tainers pots was 5.6°C higher under Mediterranean-type climate than under temperate Oceanic climate (**Table 2**), mainly due to higher average maximum temperature (+8.8°C). The average minimum daily temperatures between sites were similar (<2°C of difference), but the daily thermal amplitude was higher in Davis.

**Table 2:** Comparison of soil temperatures recorded in Rennes (Oceanic climate) and in Davis (Mediterranean-type climate) during the experiment duration (2016).

	Rennes (°C), Oceanic climate	Davis (°C), Mediterranean-type climate	Difference (°C)
Average temperature	18.1 ± 5.3	23.7 ± 7.6	5.6
Average minimum temperature	12.8 ± 3.3	14.4 ± 2.3	1.6
Average maximum temperature	25.9 ± 3.9	34.7 ± 3.4	8.8
Minimum temperature recorded	4.0	9.1	5.1
Maximum temperature recorded	33.3	39.8	6.5
Average thermal amplitude on 24 h	13.2 ± 4.2	20.4 ± 2.5	7.2
Minimum thermal amplitude on 24 h	4.3	13.2	8.9
Maximum thermal amplitude on 24 h	25.4	24.8	0.6

*Averages are expressed ± standard deviation.*

### Seed Germination, Embryo Viability, and Seedling Survivorship

#### Seed Germination

**FIGURE 1:**
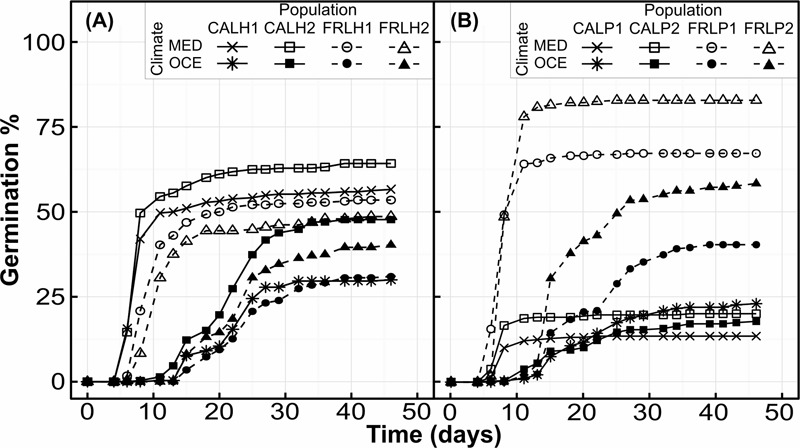
Cumulative germination percentages of *Ludwigia hexapetala* **(A)** and *Ludwigia peploides* subsp. *montevidensis* **(B)** seeds during 46 days under two contrasting climates (OCE, Oceanic climate; MED, Mediterranean-type climate), for experimental populations from two invaded ranges.

**FIGURE 2:**
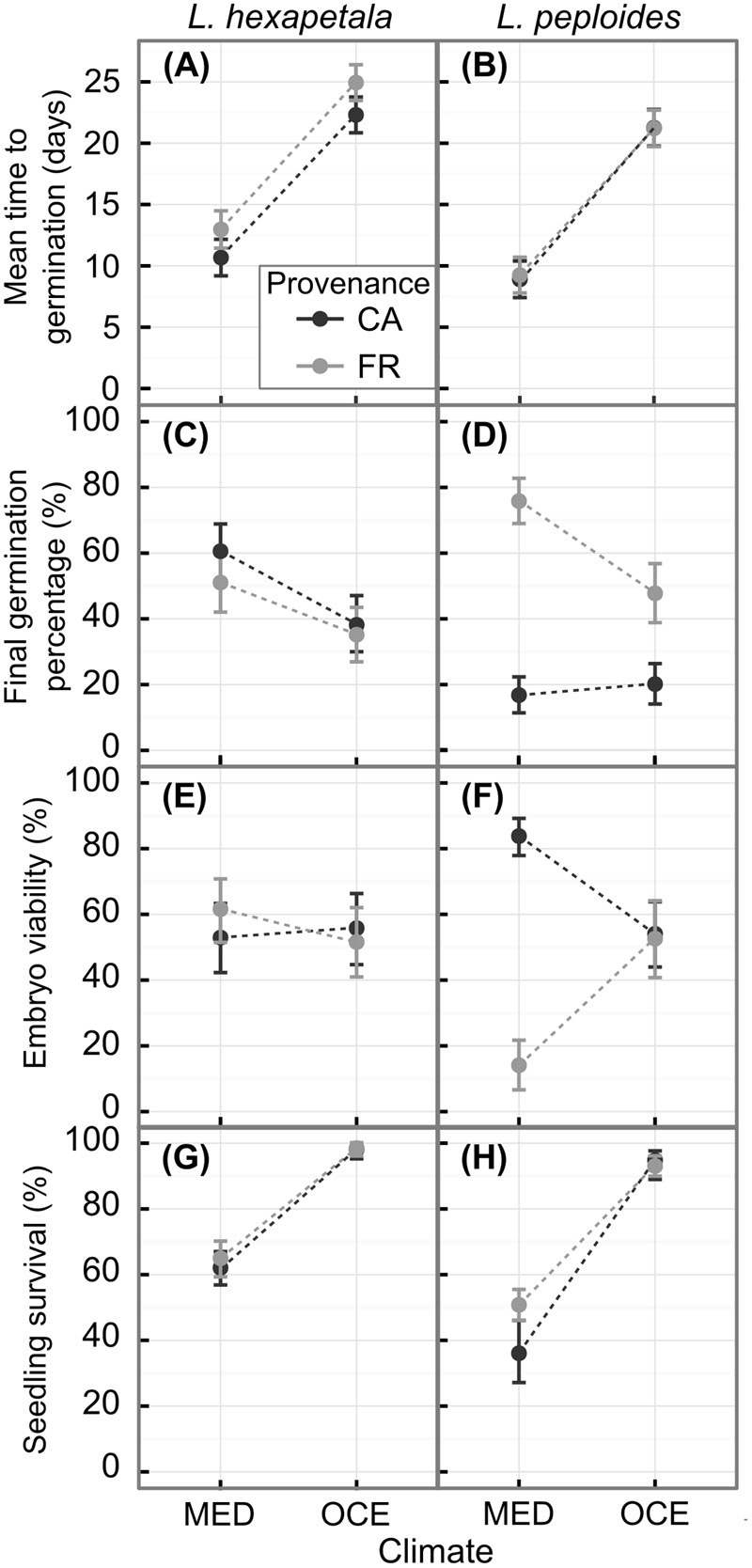
Mean time to germination, final germination percentage and percentage of viable embryos and of seedlings that survived until the end of the experiment for *Ludwigia hexapetala* **(A,C,E,G)** and *Ludwigia peploides* subsp. *montevidensis* **(B,D,F,H)** from two invasive ranges (CA, California; FR, France) and exposed to two contrasting climates [MED, Mediterranean-type climate at Davis (CA, United States); OCE, Oceanic climate at Rennes (Brittany, FR)]. The points represent predictions from linear or generalized linear models with mixed effects, and the error bars represent confidence intervals.

Temperature had a highly significant effect on MTG for both species (**Table 3**), seeds germinated about two-fold faster under warmer conditions (**Figures 1, 2A,B**). There was a significant interaction between seed provenance and species for MTG (**Table 3**); seed provenance had only a significant effect on MTG for *L. hexapetala* (**Figure 2A**). For this species, populations from France germinated 2.6 days later than populations from California, independently of climate (**Figure 1**). The linear model explained about 75% of the MTG (Supplementary Table S1).

**Table 3:** Analysis of deviance results for mean time to germination (MTG), final germination percentage, embryo viability, and seedling survival from populations of *Ludwigia hexapetala* and *Ludwigia peploides* subsp. *montevidensis* from two invaded ranges exposed to two contrasting climates.

	MTG			Final germination %		
	χ^2^	*df*	*p*	χ^2^	*df*	*p*
Climate	508.3	1	<0.001	119.6	1	<0.001
Seed provenance	6.0	1	0.014	21.2	1	<0.001
Species	23.3	1	<0.001	3.0	1	0.082
Climate:seed provenance	0.1	1	0.98	12.9	1	<0.001
Climate:species	0.1	1	0.74	2.3	1	0.13
Seed provenance:species	4.8	1	0.029	39.6	1	<0.001
Climate:seed provenance:species	0.1	1	0.73	42.2	1	<0.001

	**Embryo viability**			**Seedling survival**		
	**χ^2^**	***df***	***p***	**χ^2^**	***df***	***p***

Climate	16.5	1	<0.001	230.6	1	<0.001
Seed provenance	6.4	1	0.011	3.6	1	0.059
Species	0.02	1	0.88	38.3	1	<0.001
Climate:seed provenance	17.9	1	<0.001	1.9	1	0.17
Climate:species	1.2	1	0.26	1.3	1	0.25
Seed provenance:species	14.1	1	<0.001	2.1	1	0.15
Climate:seed provenance:species	74.9	1	<0.001	1.1	1	0.29

The final germination percentage was impacted by climate, seed provenance, the interaction between the two factors, and some of their interaction with species effect (**Table 3**). The generalized linear model performed explained 19% of the final germination percentage (Supplementary Table S2). Seeds of *L. hexapetala* from California germinated 42% more when exposed to warmer temperature, while there was no significant climate effect for seeds of *L. hexapetala* from France, only a tendency (**Figures 2C,D**). The higher temperatures under Mediterranean-type climate favored the germination percentage of the seeds of *L. peploides* from France, with on average 3.3-fold higher than when exposed to temperate Oceanic climate temperatures. However, the germination of seeds of *L. peploides* from California did not change with temperature; their germination percentage was similar under both climate conditions. The geographical origin of the seeds had an impact on the germination percentage only for populations of *L. peploides*: seeds from France germinated 4.5-fold higher than those from California when exposed to Mediterranean-type climate and 2.4-fold higher when exposed to Oceanic climate (**Figure 2D**).

#### Embryo Viability

There were significant interactions between climate and seed provenance, between seed provenance and species, and between climate, seed provenance, and species for the embryo viability (**Table 3**). The generalized linear model explained 19.5% of the embryo viability (Supplementary Table S2). There was no effect of seed provenance or of climate on the embryos viability of *L. hexapetala*, with an average of 55 ± 4% of embryos viable at the end of the experiment (**Figure 2E**). However, for *L. peploides*, the embryo viability was 5.9-fold higher for seeds from California than for seeds from France when exposed to the temperatures of the Mediterranean-type climate. When exposed to the temperatures of Oceanic climate, the embryo viability of *L. peploides* was 1.5-fold lower than in Mediterranean-type climate for seeds from California, and 3.7-fold higher for seeds from France (**Figure 2F**).

#### Seedling Survivorship

Climate and species had a significant effect on seedling survivorship (**Table 3**). For both species, the survival percentage of seedlings was higher under Oceanic climate than under Mediterranean-type climate, 1.5-fold higher for *L. hexapetala* and 2.2-fold higher for *L. peploides* (**Figures 2G,H**). The generalized linear model performed explained about 45% of seedling survivorship (Supplementary Table S2).

### Seedling Growth

**FIGURE 3:**
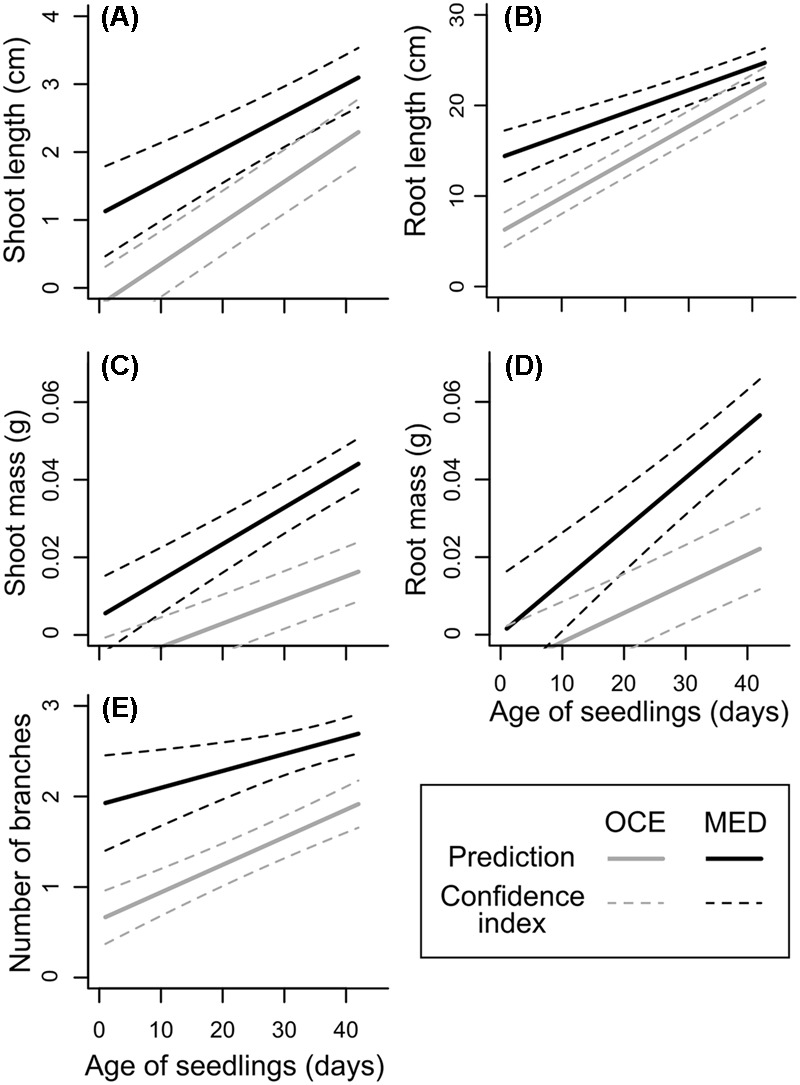
Predictions from linear models with mixed effects for shoot length **(A)**, root length **(B)**, shoot mass **(C)**, root mass **(D)**, and number of branches **(E)** depending on seedling age for *Ludwigia peploides* subsp. *montevidensis* from France exposed to two contrasting climates (OCE, Oceanic climate; MED, Mediterranean-type climate). Results for *L. peploides* from California and for *L. hexapetala* are shown in Supplementary Figure S2.

The linear model with mixed effects explained 46–73% of the variability of the length and mass of shoots and roots, and of the number of branches, including 8–16% due to capsule and population effects (Supplementary Table S3). For the five variables, seedling age explained most of the variability (**Table 4**) as the size and mass of shoots and roots as well as the number of branches increased with age of seedlings (**Figure 3**). There was a significant interaction between climate and seedling age for root length and mass and for shoot mass (**Table 4**). Indeed, initially the roots were about two-fold longer when seedlings were exposed to the warmer climate, but this difference disappeared when they got older. In contrast, there was no difference due to climate for shoot and root mass when seedlings were a few days old, but when seedlings aged, their biomass increased significantly more when they grew in a warmer climate, up to four- and three-fold heavier for shoots, respectively (**Figures 3C,D**). Consistent with this result, the number of branches was about twice more important when seedlings grew under Mediterranean-type climate (**Table 4** and **Figure 3E**). These patterns were observed in French and Californian naturalized provenances of *L. peploides* and of *L. hexapetala* (Supplementary Figure S2). The effect of the range where the capsules were collected influenced the number of branches, with more branches for population from France. Results also showed that there was an effect of the interaction between climate and species on shoot and root length and on shoot mass (**Table 4**).

**Table 4:** Analysis of deviance results for shoot length (cm), root length (cm), shoot mass (g), root mass (g), and number of branches of seedlings from populations of *Ludwigia hexapetala* and *Ludwigia peploides* subsp. *montevidensis* from two invaded ranges exposed to two contrasting climates.

	Shoot length			Shoot mass			Number of branches		
	χ^2^	*df*	*p*	χ^2^	*df*	*p*	χ^2^	*df*	*p*
Seedlings age	291.8	1	<0.001	197.0	1	<0.001	70.4	1	<0.001
Climate	7.4	1	0.006	16.4	1	<0.001	22.2	1	<0.001
Seed provenance	1.9	1	0.16	2.9	1	0.088	24.4	1	<0.001
Species	6.2	1	0.012	19.9	1	<0.001	22.1	1	<0.001
Seedling age:climate	2.8	1	0.11	8.5	1	<0.001	2.4	1	0.12
Seed provenance:climate	0.5	1	0.50	0.1	1	0.72	2.9	1	0.089
Species:climate	10.9	1	<0.001	32.3	1	<0.001	2.5	1	0.11

	**Root length**			**Root mass**					
	**χ^2^**	***df***	***p***	**χ^2^**	***df***	***p***			

Seedling age	500.5	1	<0.001	126.5	1	<0.001			
Climate	3.9	1	0.049	26.7	1	<0.001			
Seed provenance	0.06	1	0.79	0.5	1	0.48			
Species	4.1	1	0.043	0.3	1	0.58			
Seedlings age:climate	14.5	1	<0.001	10.2	1	<0.001			
Seed provenance:climate	3.3	1	0.07	2.9	1	0.087			
Species:climate	47.7	1	<0.001	0.05	1	0.82			

## Discussion

**FIGURE 4:**
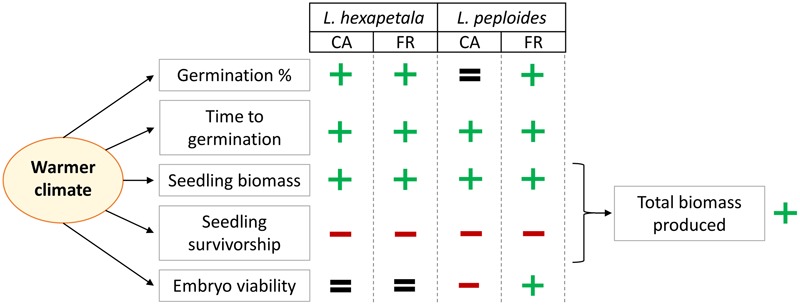
Summary of our results about the effect of warmer temperature on the germination percentage, the mean time to germination, the seedling biomass, the seedling survivorship, and on the embryo viability (after 46 days of exposure) of *Ludwigia hexapetala* and *Ludwigia peploides* subsp. *montevidensis* from two invasive ranges (CA, California; FR, France). The plus sign (+) indicates that the warmer climate had a positive impact on the variable, the minus sign (–) represents a negative effect of temperature increase, and the equals sign (=) indicates that higher temperature did not generate significant changes on the tested variable.

In this experiment, we examined the germination capacity and seedling growth of two invasive species exposed to the distinctive temperature conditions of two non-native regions. We demonstrated that the average difference in temperature of 6°C between the two sites during the experiment had an impact on tested variables (MTG, final germination percentage, seedling survivorship, seedling characteristics, and the embryo viability at the end of the experiment), depending on species and on the origin of the seeds. The results obtained are summarized in **Figure 4**. To our knowledge there is no existing study that consider the effect of temperature increase on the germination and seedling growth of macrophytes in and from multiple invaded ranges.

### Temperature Effect

We demonstrated that, for the two tested regions, higher air and soil temperature induced greater performances for seed germination and seedling biomass, but also increased seedling mortality for the tested populations of *L. hexapetala* and *L. peploides*. We had hypothesized that such temperature conditions would have improved performances of all tested variables, so our first hypothesis is partially validated. Nonetheless, the lower number of seedlings that survived under hotter Mediterranean-type climate conditions was counteracted by the higher germination percentage of some populations under these conditions, and above all by the higher biomass of seedlings shoots and roots. Indeed, the product of final germination percentage, seedling survivorship percentage, and of the mean total biomass per seedling showed that the total biomass produced from *Ludwigia* seeds was on average six-fold greater under the warmer climate (Supplementary Table S4). If, according to Tivy (1990), decrease in warmth of the growing season with increasing latitude is compensated by longer day length, in our experiment the slightly higher day length in Rennes did not counterbalance the effects of lower temperatures in that range. Bittencourt et al. (2017) suggest *Eragrostis plana* may germinate better at fluctuating temperature because daily temperature oscillation is deeply involved in the transcriptional regulation of a set of enzymes regulating two hormones essential to the germination process. Thus, higher levels of germination under the Mediterranean-type climate could also be explained by the greater daily thermal amplitude in that range. The seeds germinated sooner when exposed to warmer conditions, a result comparable to that found by Gillard et al. (2017b) for the studied species. Earlier germination under increased temperature may represent a competitive advantage for invasive water primroses over later germinating species. Seedlings from both species had greater shoot and root biomass when grown under higher temperature. This is consistent with the results of Yen and Myerscough (1989) who showed that *L. peploides* had greater biomass when grown at 40°C, a temperature close to the maximum temperature recorded in Davis (Mediterranean-type climate) during our experiment. However, the maximum length of shoots and roots was not different depending on climatic conditions. Thus, the greater biomass of the seedlings when grown in warmer conditions is rather due to the larger number of stems, and probably of roots, as well as to their thickness or to the size of leaves.

We showed that the percentage of seedling survivorship of both study species decreased when they were exposed to maximum temperatures above 34°C, independently of their provenance. Most seedling mortality occurred before 2 weeks following seedling emergence. Kolb and Robberecht (1996) showed that water transport through seedling stems in *Pinus ponderosa* seedlings can act as a heat transfer mechanism and protect the plant from high temperature. Failure to survive may have been due to insufficient root development of seedlings to ensure a transpiration rate to survive a lethal threshold temperature. While seedling mortality was higher under the Mediterranean-type temperatures, it is possible that under the same conditions most seedlings of native species from an Oceanic climate would be even less heat tolerant, as shown by Hou et al. (2014) on herb species. On the other hand, a 2°C warming applied to four grassland species in Australia led to less seed germination and seedling establishment for the two invasive species compared to natives (Williams et al., 2007). Therefore, any statement about the importance of regeneration from seeds of *L. hexapetala* and *L. peploides* on native communities should also consider the germination, seedling growth and competitive ability of the native species, which would require further investigation. The greater growth and biomass production of plants generated from sexual propagules of the two water primroses provide evidence that suggests the regeneration of these invasive species from sexual reproduction might be enhanced by increased temperature. Thus, in invasive ranges with Oceanic climates, under future warmer climates, the invasiveness of the two water primroses may be expected to increase. Implementation of protocols for the monitoring and management of seed banks in invaded ranges could contribute to limit further colonization by these species and management for conservation of native biodiversity. Our experiment does not allow us to draw conclusions about the impact of temperature increase in regions where temperatures are already high. Nonetheless, one can expect that the seedling mortality of water primrose species may rise considerably, leading to lower biomass production from sexual propagules.

### Impact of Seed Provenance

Using reciprocal transplants of seeds into outdoor mesocosms, we showed that seed provenance had an influence on germination percentage and velocity, on embryo viability, and on the number of branches produced, depending on species. We had hypothesized that seed germination and seedling performance would vary depending on their provenance, so our second hypothesis is partly validated. In contrast to our results regarding seedling survivorship, Gross et al. (2016) found that non-local provenance seeds of *Hardenbergia violacea* performed poorly to local climate in Australia, and that most survivors were from local provenances. One of the strongest impact of seed provenance was on the embryo viability of *L. peploides*, for which the proportion of viable embryos decreased when seeds were not grown in the range where collected. Nonetheless, the low number of seeds from France still viable at the end of the experiment under Mediterranean-type climate is directly related to the higher germination percentage of these populations under this warmer climate. Indeed, most of the seeds with viable embryos had germinated under these conditions, while fewer seeds exposed to lower temperature had germinated despite their viability. On the contrary, as the germination percentages of *L. peploides* from California were similar when exposed to the two temperature regimes, the lower proportion of viable embryos implies that the exposure of seeds to colder temperature decreased their viability. To date and to our knowledge, the sole results about embryo viability of water primroses are those of Ruaux et al. (2009), who showed that only negative temperature (-15°C) in humid conditions decreased the embryo viability, for populations from France.

Populations of *L. peploides* from northwestern France germinated better than those from California regardless of temperature. This could be due to many factors, such as genetic differences, differences in phenotypic plasticity, environmental characteristics in the source population sites, or to the climatic conditions during the fruit maturation (Donohue et al., 2010; Walck et al., 2011). In view of our results, this last hypothesis would signify that *L. peploides* produces more viable seed when parents experience relatively mild summer temperature, as in Oceanic climates. Moreover, the differences observed in this experiment between the two ranges for *L. peploides* may be due to the introduction of plants originated from different native populations and differing by their genotype. The seed viability tested at the end of our experiment might have been influenced by the temperature to which they were exposed during 46 days, thus it would be necessary to test the viability of seeds of the two species before any treatment. We showed that the germination capacity and the seedling growth of *L. hexapetala* and *L. peploides* were rather dependent on temperature applied on seeds and seedlings than on temperature during fruit maturation; our second hypothesis is therefore rejected. Further investigation would be needed in order to control the maternal effects between these populations, by growing plants from seeds for two generations under the same environmental conditions (Fenster and Galloway, 2000). Future research comparing germination dynamics of seeds of the two water primroses from their native range, tested in native and exotic range conditions, could yield valuable information about the evolution of germination capacity (Hierro et al., 2009; Gioria and Pyšek, 2017; Udo et al., 2017).

### Species Effect

The seedling survivorship of *L. peploides* was lower than that of *L. hexapetala* under warm conditions. For some characteristics, the seedlings of the polyploid *L. hexapetala* were affected by temperature for a shorter time than the diploid *L. peploides*, but for other characteristics it is the diploid that showed the least differences between the two temperature regimes. Furthermore, on average, seedlings of *L. peploides* produced greater biomass during this initial growth stage than seedlings of *L. hexapetala*, especially under warm conditions, with longer shoots and roots, heavier shoots, and more branches. Regarding the percentages of germination and of viable seeds, the differences between the two species were strongly dependent on the origin of the seeds. Thus, in warm conditions, seedling survivorship of the polyploid species *L. hexapetala* was greater than those of the diploid *L. peploides*, but showed lower growth performances than those of *L. peploides*. The higher ploidy level of *L. hexapetala* does not confer it overall better germination and seedling performances compared to *L. peploides*, for the tested variables; our third hypothesis is rejected. This is comparable with the findings of Grewell et al. (2016) who showed that asexual ramets of *L. peploides* outperform *L. hexapetala* at the early stage of growth, with higher biomass production and higher growth rate suggesting alternate growth strategies for establishment and colonization. Seedlings of both species prioritized the allocation of energy to roots before the growth of shoots, regardless of range or climate, a classic pattern in early plant development (Weiner, 2004). Such a strategy ensures the uptake of nutrients and water for rapid growth, and allows the anchorage of the individuals.

## Conclusion

In this study, we compared the germination capacity and the seedling growth of two congeneric invasive macrophytes from two invasive ranges under contrasting climates. We showed that the effect of climate conditions on germination percentage and on seed viability depended on species and on seed origin. When seedlings emerged and initial growth was at higher temperatures, their survivorship decreased, but their biomass increased significantly, enough to widely counterbalance seedling mortality. In the context of climate warming, our results suggest that the invasiveness of water primroses in ranges with temperate Oceanic climates may increase as global temperatures rise. The relatively high germination capacity and biomass production from sexual propagules of water primroses represents a potential threat for native biodiversity, especially in communities with low taxonomic diversity among native species, which have been shown to be less resistant to invasion (Gallien and Carboni, 2017). In this experiment, characteristics of early germinants that survive may be a bit different than characteristics of later germinants, that may need to be accounted for in a refinement of a model on seedling responses to increasing temperature. The difference of ploidy between the two species was not always associated with consistent differences in their performance relative to temperature regime. To determine the success of survival of seedlings emergent from seed banks compared to those of vegetative fragments, both types of propagules should be grown under the same conditions. Given the high germination percentages of *L. hexapetala* from California and *L. peploides* from northwestern France, in addition to the control of vegetative stems (manual and mechanical removal, herbicides), managers might want to induce management early before development of ripe seeds of these species to impede their dispersal and colonization in the two ranges and thereby preserve native biodiversity. As global temperatures rise, long-term management will be required to address secondary invasion of water primroses from seed bank emergence to restore biodiversity and aquatic habitats dominated by these highly invasive macrophyte species.

## Author Contributions

BG, CF, GT, and MG designed the experiment. MG conducted the experiments, with help from BG, CD, CF, and GT. MG analyzed the data and wrote the manuscript with contributions from all the authors.

## Conflict of Interest Statement

The authors declare that the research was conducted in the absence of any commercial or financial relationships that could be construed as a potential conflict of interest.

## References

[B1] Barton K. (2016). *MuMIn: Multi-Model Inference.* Available at: http://cran.r-project.org/package=MuMIn

[B2] Bates D. Maechler M. Bolker B. Walker S. (2015). Fitting linear mixed-effects models using lme4. *J. Stat. Softw.* 67 1–48. 10.18637/jss.v067.i01

[B3] Benaglia T. Chauveau D. Hunter D. R. Young D. S. (2009). mixtools: an R package for analyzing mixture models. *J. Stat. Softw.* 32 1–29. 10.18637/jss.v032.i06

[B4] Bittencourt H. V. H. Bonome L. T. S. Trezzi M. M. Vidal R. A. Lana M. A. (2017). Seed germination ecology of Eragrostis plana, an invasive weed of South American pasture lands. *S. Afr. J. Bot.* 109 246–252. 10.1016/j.sajb.2017.01.009

[B5] Dandelot S. Matheron R. Le Petit J. Verlaque R. Cazaubon A. (2005). Temporal variations of physicochemical and microbiological parameters in three freshwater ecosystems (southeastern France) invaded by Ludwigia spp. *C R Biol.* 328 991–999. 10.1016/j.crvi.2005.09.007 16286088

[B6] Donohue K. Rubio de Casas R. Burghardt L. Kovach K. Willis C. G. (2010). Germination, postgermination adaptation, and species ecological ranges. *Annu. Rev. Ecol. Evol. Syst.* 41 293–319. 10.1146/annurev-ecolsys-102209-144715

[B7] Evangelista H. B. A. Thomaz S. M. Umetsu C. A. (2014). An analysis of publications on invasive macrophytes in aquatic ecosystems. *Aquat. Invasions* 9 521–528. 10.3391/ai.2014.9.4.10

[B8] Fenster C. B. Galloway L. F. (2000). Inbreeding and outbreeding depression in natural populations of *Chamaecrista fasciculata* (Fabaceae). *Conserv. Biol.* 14 1406–1412. 10.1046/j.1523-1739.2000.99234.x

[B9] Fox J. Weisberg S. (2011). *An R Companion to applied regression* Second Edn. Thousand Oaks, CA: Sage.

[B10] Gallardo B. Clavero M. Sánchez M. I. Vilà M. (2016). Global ecological impacts of invasive species in aquatic ecosystems. *Glob. Chang Biol.* 22 151–163. 10.1111/gcb.13004 26212892

[B11] Gallien L. Carboni M. (2017). The community ecology of invasive species: where are we and what’s next? *Ecography (Cop.).* 40 335–352. 10.1111/ecog.02446

[B12] Gillard M. AThiébaut G. Deleu C. Leroy B. (2017a). Present and future distribution of three aquatic plants taxa across the world: decrease in native and increase in invasive ranges. *Biol. Invasions* 19 2159–2170. 10.1007/s10530-017-1428-y

[B13] Gillard M. Grewell B. J. Deleu C. Thiébaut G. (2017b). Climate warming and water primroses: germination responses of populations from two invaded ranges. *Aquat. Bot.* 136 155–163. 10.1016/j.aquabot.2016.10.001

[B14] Gioria M. Pyšek P. (2017). Early bird catches the worm: germination as a critical step in plant invasion. *Biol. Invasions* 19 1050–1088. 10.1007/s10530-016-1349-1

[B15] Grewell B. J. Skaer Thomason M. J. Futrell C. J. Iannucci M. Drenovsky R. E. (2016). Trait responses of invasive aquatic macrophyte congeners: colonizing diploid outperforms polyploid. *AoB Plants* 8:plw014 10.1093/aobpla/plw014 PMC4823780 26921139

[B16] Gross C. L. Fatemi M. Simpson I. H. (2016). Seed provenance for changing climates: early growth traits of nonlocal seed are better adapted to future climatic scenarios, but not to current field conditions. *Restor. Ecol.* 25 577–586. 10.1111/rec.12474

[B17] Haury J. Druel A. Cabral T. Paulet Y. Bozec M. Coudreuse J. (2014). Which adaptations of some invasive Ludwigia spp. (Rosidae, Onagraceae) populations occur in contrasting hydrological conditions in Western France? *Hydrobiologia* 737 45–56. 10.1007/s10750-014-1815-7

[B18] Heino J. Virkkala R. Toivonen H. (2009). Climate change and freshwater biodiversity: detected patterns, future trends and adaptations in northern regions. *Biol. Rev.* 84 39–54. 10.1111/j.1469-185X.2008.00060.x 19032595

[B19] Hierro J. L. Eren Ö Khetsuriani L. Diaconu A. Török K. Montesinos D. (2009). Germination responses of an invasive species in native and non-native ranges. *Oikos* 118 529–538. 10.1111/j.1600-0706.2009.17283.x

[B20] Hou Q.-Q. Chen B.-M. Peng S.-L. Chen L.-Y. (2014). Effects of extreme temperature on seedling establishment of nonnative invasive plants. *Biol. Invasions* 16 2049–2061. 10.1007/s10530-014-0647-8

[B21] Hoya A. Shibaike H. Morita T. Ito M. (2007). Germination characteristics of native Japanese dandelion autopolyploids and their putative diploid parent species. *J. Plant Res.* 120 139–147. 10.1007/s10265-006-0034-3 17061143

[B22] Hussner A. (2012). Alien aquatic plant species in European countries. *Weed Res.* 52 297–306. 10.1111/j.1365-3180.2012.00926.x

[B23] Hussner A. (2014). Long-term macrophyte mapping documents a continuously shift from native to non-native aquatic plant dominance in the thermally abnormal River Erft (North Rhine-Westphalia, Germany). *Limnologica* 48 39–45. 10.1016/j.limno.2014.05.003

[B24] Kolb P. F. Robberecht R. (1996). High temperature and drought stress effects on survival of *Pinus ponderosa* seedlings. *Tree Physiol.* 16 665–672. 10.1093/treephys/16.8.665 14871688

[B25] Leck M. A. Brock M. A. (2000). Ecological and evolutionary trends in wetlands: Evidence from seeds seed banks in New South Wales, Australia and New Jersey, USA. *Plant Species Biol.* 15 97–112. 10.1046/j.1442-1984.2000.00031.x

[B26] Li W. (2014). Environmental opportunities and constraints in the reproduction and dispersal of aquatic plants. *Aquat. Bot.* 118 62–70. 10.1016/j.aquabot.2014.07.008

[B27] Luque E. G. Fernández I. C. D. Mercado F. G. (2013). Effect of salinity and temperature on seed germination in *Limonium cossonianum*. *Botany* 91 12–16. 10.1139/cjb-2012-0157

[B28] Mainka S. A. Howard G. W. (2010). Climate change and invasive species: double jeopardy. *Integr. Zool.* 5 102–111. 10.1111/j.1749-4877.2010.00193.x 21392328

[B29] Mazerolle M. (2016). *AICcmodavg: Model Selection and Multimodel Inference Based on (Q)AIC(c).* Available at: http://cran.r-project.org/package=AICcmodavg

[B30] Michelan T. S. Thomaz S. M. Mormul R. P. Carvalho P. (2010). Effects of an exotic invasive macrophyte (tropical signalgrass) on native plant community composition, species richness and functional diversity. *Freshw. Biol.* 55 1315–1326. 10.1111/j.1365-2427.2009.02355.x

[B31] Nakagawa S. Schielzeth H. (2013). A general and simple method for obtaining R2 from generalized linear mixed-effects models. *Methods Ecol. Evol.* 4 133–142. 10.1111/j.2041-210x.2012.00261.x

[B32] Nicolet P. Biggs J. Fox G. Hodson M. J. Reynolds C. Whitfield M. (2004). The wetland plant and macroinvertebrate assemblages of temporary ponds in England and Wales. *Biol. Conserv.* 120 265–282. 10.1016/j.biocon.2004.03.010

[B33] Okada M. Grewell B. J. Jasieniuk M. (2009). Clonal spread of invasive *Ludwigia hexapetala* and L. *grandiflora* in freshwater wetlands of California. *Aquat. Bot.* 91 123–129. 10.1016/j.aquabot.2009.03.006

[B34] Peeters E. T. H. M. van Zuidam J. P. van Zuidam B. G. Van Nes E. H. Kosten S. Heuts P. G. M. (2013). Changing weather conditions and floating plants in temperate drainage ditches. *J. Appl. Ecol.* 50 585–593. 10.1111/1365-2664.12066

[B35] Porter R. H. Durrell M. Romm H. J. (1947). The use of 2,3,5-triphenyl-tetrazoliumchloride as a measure of seed germinability. *Plant Physiol.* 22 149–159. 10.1104/pp.22.2.149 16654086 PMC405851

[B36] R Development Core Team (2015). *R: A Language and Environment for Statistical Computing.* Vienna: R Foundation for Statistical Computing.

[B37] Ruaux B. Greulich S. Haury J. Berton J.-P. (2009). Sexual reproduction of two alien invasive Ludwigia (Onagraceae) on the middle Loire River, France. *Aquat. Bot.* 90 143–148. 10.1016/j.aquabot.2008.08.003

[B38] Silvertown J. (2008). The evolutionary maintenance of sexual reproduction: evidence from the ecological distribution of asexual reproduction in clonal plants. *Int. J. Plant Sci.* 169 157–168. 10.1086/523357

[B39] Stiers I. Crohain N. Josens G. Triest L. (2011). Impact of three aquatic invasive species on native plants and macroinvertebrates in temperate ponds. *Biol. Invasions* 13 2715–2726. 10.1007/s10530-011-9942-9

[B40] Strayer D. L. (2010). Alien species in fresh waters: ecological effects, interactions with other stressors, and prospects for the future. *Freshw. Biol.* 55 152–174. 10.1111/j.1365-2427.2009.02380.x

[B41] Thouvenot L. Haury J. Thiebaut G. (2013). A success story: water primroses, aquatic plant pests. *Aquat. Conserv. Mar. Freshw. Ecosyst.* 23 790–803. 10.1002/aqc.2387

[B42] Tivy J. (1990). *Agricultural Ecology.* Ann Arbor, MI: University of Michigan.

[B43] Udo N. Tarayre M. Atlan A. (2017). Evolution of germination strategy in the invasive species Ulex europaeus. *J. Plant Ecol.* 10 375–385. 10.1093/jpe/rtw032

[B44] Vickery R. K. (1967). Ranges of temperature tolerance for germination of mimulus seeds from diverse populations. *Ecology* 48 647–651. 10.2307/1936508

[B45] Vilà M. Basnou C. Pyšek P. Josefsson M. Genovesi P. Gollasch S. (2010). How well do we understand the impacts of alien species on ecosystem services? A pan-European, cross-taxa assessment. *Front. Ecol. Environ.* 8:135–144. 10.1890/080083

[B46] Vojtkó A. E. Mesterházy A. Süveges K. Valkó O. Lukács B. A. (2017). Changes in sediment seed-bank composition of invaded macrophyte communities in a thermal river. *Freshw. Biol.* 62 1024–1035. 10.1111/fwb.12922

[B47] Wagner W. L. Hoch P. C. Raven P. H. (2007). Revised classification of Onagraceae. *Syst. Bot. Monogr.* 83 1–222. 10.11646/zootaxa.3672.1.1

[B48] Walck J. L. Hidayati S. N. Dixon K. W. Thompson K. Poschlod P. (2011). Climate change and plant regeneration from seed. *Glob. Chang Biol.* 17 2145–2161. 10.1111/j.1365-2486.2010.02368.x

[B49] Weiner J. (2004). Allocation, plasticity and allometry. *Perspect. Plant Ecol. Evol. Syst.* 6 205–206. 10.1078/1433-8319-00083

[B50] Williams A. L. Wills K. E. Janes J. K. Vander Schoor J. K. Newton P. C. D. Hovenden M. J. (2007). Warming and free-air CO2 enrichment alter demographics in four co-occurring grassland species. *New Phytol.* 176 365–374. 10.1111/j.1469-8137.2007.02170.x 17888117

[B51] Yarrow M. Marin V. H. Finlayson M. Tironi A. Delgado L. E. Fisher F. (2009). The ecology of *Egeria densa* Planchon (Liliopsida: Alismatales): a wetland ecosystem engineer? *Rev. Chil. Hist. Nat.* 82 299–313. 10.4067/S0716-078X2009000200010

[B52] Yen S. Myerscough P. J. (1989). Co-existence of three species of amphibious plants in relation to spatial and temporal variation: Investigation of plant responses. *Aust. J. Ecol.* 14 305–318. 10.1111/j.1442-9993.1989.tb01439.x

