# Supplementary material for: Germination and Seedling Growth of Water Primroses: A Cross Experiment between Two Invaded Ranges with Contrasting Climates
Source: Front Plant Sci. 2017 Sep 26;8:1677. doi: 10.3389/fpls.2017.01677 (PMC5623178; doi:10.3389/fpls.2017.01677)
Supplement: Supplementary file 1 [file Data_Sheet_1.pdf]

Germination and seedling growth of water primroses: a cross experiment between two invaded ranges with contrasting climates

Supplementary Material

Morgane Gillard, Brenda J. Grewell, Caryn J. Futrell, Carole Deleu, Gabrielle Thiébaut*

***Correspondence:** Corresponding Author: gabrielle.thiebaut@univ-rennes1.fr

Supplementary material 1 – Contrasting climate between the two invasive ranges: a Mediterranean-type climate and an Oceanic climate

Figure S1. Climatic diagrams of Davis in California, USA (A) and Rennes in France (B), representing the local climate (Mediterranean-type climate and Oceanic climate, respectively). Light blue bars represent the monthly rainfall in mm, the lines depict the average high (in red) and low (in blue) temperature per month, in Celsius degree (data Source: Météo-France and USclimatedata.com).

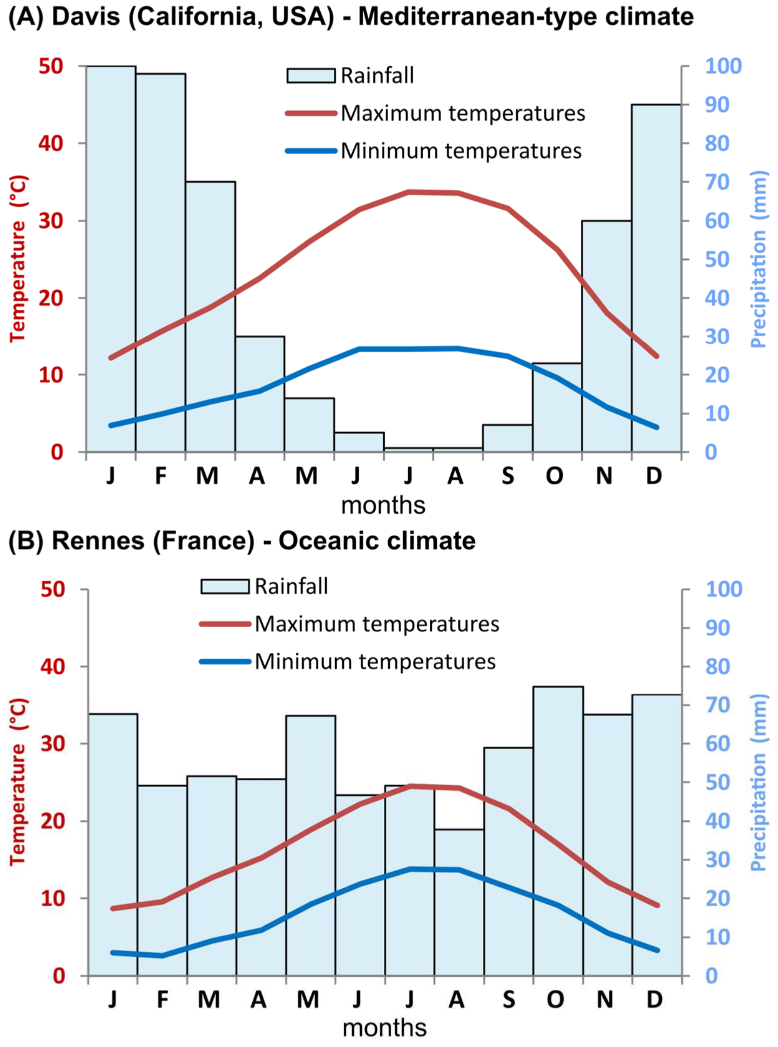

Supplementary material 2 – Proportion of variability of models explained

Table S1. Proportion of the variability explained by fixed effects (climate, range and species) and random effects (population) of the linear models applied to mean time to germination data.

	Mean time to germination
**Fixed effects**	**74.8%**
**Random effects**	**0%**
**Fixed + random effects**	**74.8%**

Table S2. Proportion of the variability explained by fixed effects (climate, range and species) and random effects (population) of the generalized linear models for binomial data.

	Seed germination	Seedling survivorship	Embryo viability
**Fixed effects**	**17.7%**	**44.8%**	**18.1%**
**Random effects**	**1.4%**	**0**	**1.4%**
**Fixed + random effects**	**19.1%**	**44.8%**	**19.5%**

Table S3. Proportion of the variability explained by fixed effects (seedling age, climate, range and species) and random effects (tank, capsule and population) of the models for seedling characteristics.

	Shoot length	Root length	Shoot weight	Root weight	Number of branches
**Fixed effects**	**47%**	**52%**	**59%**	**55%**	**38%**
**Random effects**	**16%**	**9%**	**14%**	**13%**	**8%**
**Fixed + random effects**	**63%**	**61%**	**73%**	**68%**	**46%**

Supplementary material 3 – Model results on seedling characteristics

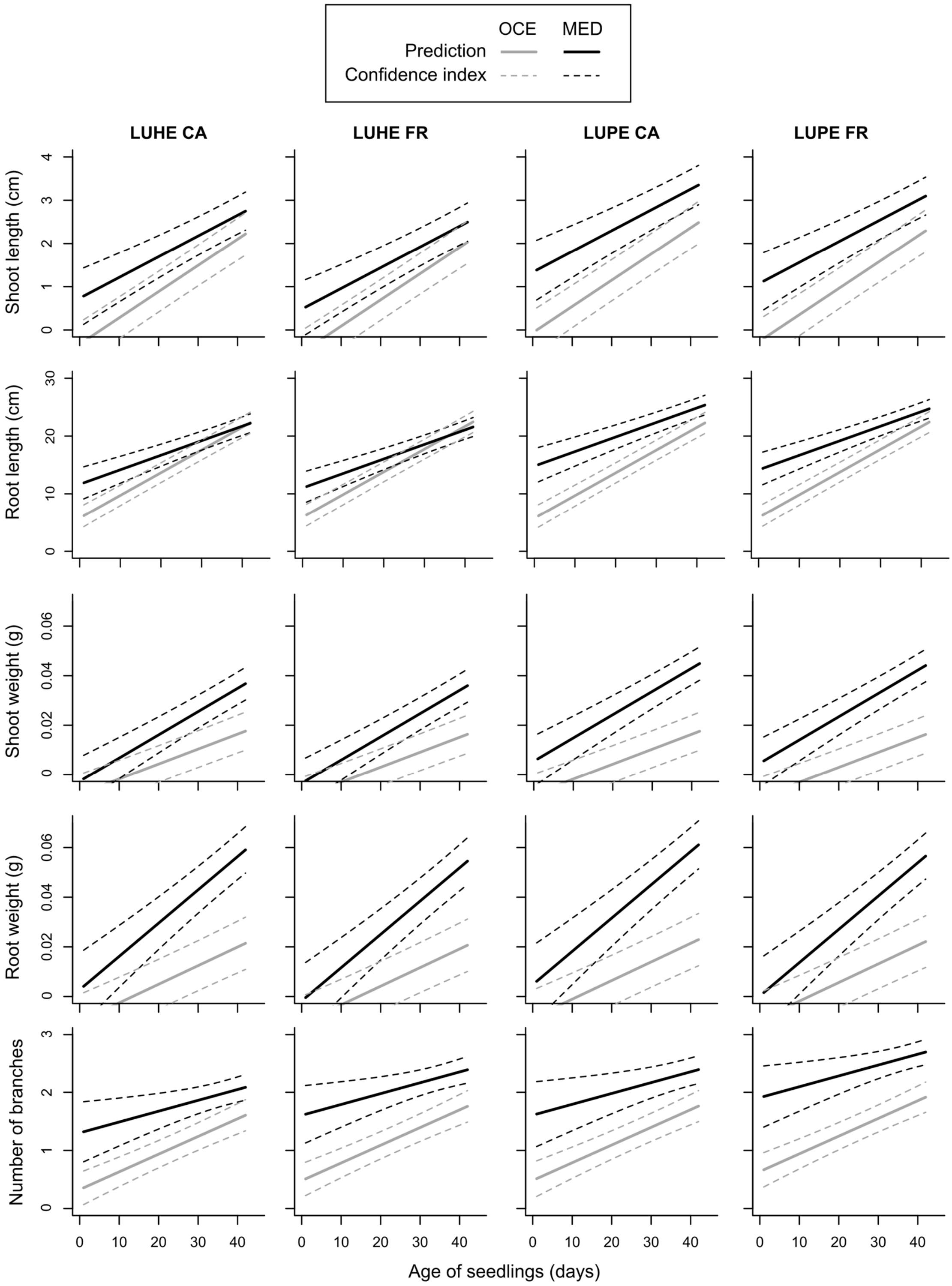

Figure S2. Predictions from linear models with mixed effects of seedling characteristics, depending on their age, for *Ludwigia hexapetala* and *Ludwigia peploides* subsp. *montevidensis* from two invasive range and exposed to two contrasting climates (OCE=Oceanic climate, MED=Mediterranean-type climate).

Supplementary material 3 – Model results for seedling characteristics

Table S4. Calculation of total seedling biomass produced under the two climates (MED= Mediterranean-type climate, OCE= Ocean climate).

Population	Climate	% germination		% survivor-ship		Mean total dry biomass per seedling (g)	/100 =	Total biomass produced (g)	Ratio MED/OCE
CALH1	MED	56.6	x	79.9	x	0.090		4.07	13.7
	OCE	30.0		99.3		0.010		0.30	
CALH2	MED	64.2		74.3		0.075		3.58	4.2
	OCE	47.7		99.3		0.018		0.85	
FRLH1	MED	53.5		81.6		0.089		3.89	11.4
	OCE	31.0		100		0.011		0.34	
FRLH2	MED	48.3		83.0		0.076		3.05	5.8
	OCE	40.3		100		0.013		0.52	
CALP1	MED	13.5		54.8		0.123		0.91	2.3
	OCE	23.1		99.0		0.017		0.39	
CALP2	MED	20.1		83.7		0.109		1.83	4.7
	OCE	17.9		99.3		0.022		0.39	
FRLP1	MED	67.4		66.0		0.095		4.23	7.1
	OCE	40.5		98.3		0.015		0.60	
FRLP2	MED	83.0		60.4		0.082		4.11	4.2
	OCE	58.5		98.6		0.017		0.98	
									Average 6.7
